# Improving Diagnostic Accuracy of Myalgic Encephalomyelitis/Chronic Fatigue Syndrome Through a Point-of-Care Clinical Algorithm

**DOI:** 10.3390/ijerph23040460

**Published:** 2026-04-03

**Authors:** Jaime Seltzer, Stephanie L. Grach, Scott D. Eggers, Melissa M. Redetzke, Katie J. Mau, Tony Y. Chon, Ravindra Ganesh

**Affiliations:** 1 The Myalgic Encephalomyelitis Action Network, Santa Clara, CA 90403, USA; jaime@meaction.net; 2 Division of Internal Medicine, Mayo Clinic, Rochester, MN 55905, USA; 3 Department of Neurology, Mayo Clinic, Rochester, MN 55905, USA; 4 Health Education and Content Services, Mayo Clinic, Rochester, MN 55905, USA; 5 Center for Digital Health, Mayo Clinic, Rochester, MN 55905, USA; 6 Baptist Health Medical Group, Baptist Health Quality Network, Miami, FL 33143, USA

**Keywords:** myalgic encephalomyelitis/chronic fatigue syndrome, quality improvement, infection-associated chronic illness, diagnostic accuracy

## Abstract

**Highlights:**

**Public health relevance—How does this work relate to a public health issue?**

- ME/CFS is a common, debilitating disease affecting millions in the United States.
- Prevalence has increased by several orders of magnitude due to COVID-19, yet the majority of patients remain undiagnosed.

**Public health significance—Why is this work of significance to public health?**

- Accessible, point-of-care clinical tools can significantly improve diagnostic accuracy and decrease diagnostic delay.
- Point-of-care tools require minimal resources yet may achieve measurable improvements in clinical practice, suggesting that educational gaps drive poor diagnostic outcomes, and that targeted knowledge delivery at the point of care can efficiently address public health-scale diagnostic crises.

**Public health implications—What are the key implications or messages for practitioners, policy makers and/or researchers in public health?**

- Point-of-care clinical decision support tools improve diagnosis and management of complex chronic conditions where medical education is inadequate; healthcare systems should prioritize developing and implementing such resources for poorly understood, prevalent diseases.
- Involving people with lived experience as co-creators of clinical tools and educational resources is essential for developing effective interventions, particularly for complex chronic conditions where clinical expertise alone may not capture critical diagnostic and management insights.

**Abstract:**

Despite the increasing prevalence and median severity of myalgic encephalomyelitis/chronic fatigue syndrome (ME/CFS), medical education on the disease is scant, leading to a diagnostic crisis in which the majority of people with ME/CFS are undiagnosed. We created a care process algorithm in AskMayoExpert accessible to all Mayo Clinic medical providers as a source for information on diagnosis and management of ME/CFS. To evaluate whether the algorithm was associated with improved diagnostic accuracy, we compared concordance before versus after the algorithm was introduced, where concordance was defined as agreement between an appropriately coded referral to Mayo Clinic’s Chronic Fatigue Specialty Clinic and the specialty clinic with an expert diagnosis of ME/CFS. Referrals to the Chronic Fatigue Specialty Clinic increased overall and were more likely to show concordance between specialist diagnosis and referral after the introduction of the ME/CFS AskMayoExpert algorithm. Particularly in diseases that are prevalent and poorly understood, a point-of-care clinical tool may offer just-in-time opportunities to improve diagnosis and management.

## 1. Introduction

Myalgic encephalomyelitis/chronic fatigue syndrome (ME/CFS) is a multisystem neurological disease [1] triggered by infection up to 80% of the time [2]. As an infection-associated chronic illness, ME/CFS may follow infection from multiple pathogens and other immunological perturbations [2,3]. Pre-pandemic, it was estimated that approximately 2.5 million people in the United States lived with ME/CFS [2]. Studies show that approximately half of people with Long COVID meet diagnostic criteria for ME/CFS at six months [4,5]. Therefore, the number of people with ME/CFS in the United States has increased substantially, with new incidence cases of ME/CFS 15 times higher than pre-pandemic levels [5]. In addition, an estimated 29% of people living with ME/CFS experience five or more years of diagnostic delay, in part because there is no single biomarker that provides a definitive diagnosis; patients’ symptom pictures vary; symptoms wax and wane; and some aspects of ME/CFS presentation may overlap with that of other diseases [2].

Studies have found that people living with ME/CFS have a lower quality of life than those with chronic renal failure, stroke, prostate cancer, congestive heart failure, and multiple sclerosis [6,7,8]. Although there is under-ascertainment in ethnic and racial minorities, ME/CFS is a common and debilitating disease that affects people of all ages, genders, races, and socioeconomic backgrounds [2,9,10,11]. ME/CFS cost the United States an estimated $17–24 billion dollars in 2008 yet remains more underfunded with respect to disease burden than any condition monitored by the National Institutes of Health [12,13]. Moreover, few major medical centers have programs dedicated to the evaluation and management of patients with chronic complex, often post-viral disease.

Mayo Clinic General Internal Medicine has provided direct patient care to those with these conditions. Shortly after the recognition of SARS-CoV-2, Mayo developed a specialized clinic to care for patients with Long COVID. However, the number of Long COVID patients who would benefit from expert assessment far exceeds current capacity. As the number of patients with infection-associated chronic disease rises due to SARS-CoV-2, it is increasingly important for all practitioners to familiarize themselves with infection-associated chronic conditions including ME/CFS and Long COVID. This is especially true in primary care settings, though it is also common for patients to present to subspecialty clinics including pulmonary or cardiology.

**Figure 1:**
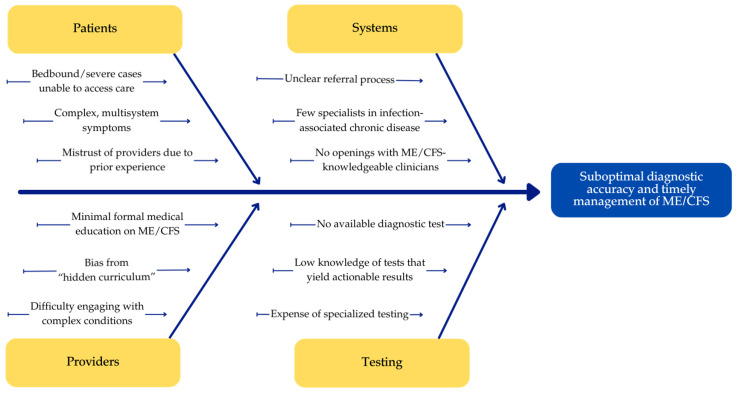
Root cause analysis covering common contributors to suboptimal diagnostic accuracy and timely management of ME/CFS.

Despite guideline and coding changes, general understanding of infection-associated chronic conditions remains poor [2,14,15]. Medical education in ME/CFS has been scant despite its prevalence and severity: only one in three medical schools touch on the disease, and it is mentioned in fewer than half of medical school textbooks [16,17]. This has led to a diagnostic crisis in which the vast majority of people living with ME/CFS are undiagnosed [11,18]. Potential root causes of suboptimal diagnostic accuracy and timely management are shown in Figure 1. Better tools are needed to increase diagnostic accuracy and introduce best practice treatment protocols in order to better serve people with ME/CFS at Mayo Clinic. Our team developed a point-of-care clinical care algorithm through AskMayoExpert (AME) to serve this need, including specialist clinicians and researchers with lived experience.

The aim of this study was to assess the impact of a newly created care process algorithm, “Myalgic Encephalomyelitis/Chronic Fatigue Syndrome”, on diagnostic concordance of patients referred to the Mayo Clinic–Rochester Chronic Fatigue Clinic for suspected myalgic encephalomyelitis/chronic fatigue syndrome. To evaluate whether the algorithm was associated with improved diagnostic accuracy, we compared concordance before versus after the algorithm was introduced, where concordance was defined as agreement between referral to Mayo Clinic’s Chronic Fatigue Specialty Clinic and the specialty clinic’s expert diagnosis of ME/CFS. We hypothesized that, through development of the clinical decision support tool via AskMayoExpert, concordance would increase between referral to the Mayo Clinic Chronic Fatigue Syndrome Clinic and specialist diagnosis of myalgic encephalomyelitis/chronic fatigue syndrome.

## 2. Materials and Methods

### 2.1. Intervention Development

AskMayoExpert is an online point-of-care clinical knowledge resource actively utilized by over 30,000 healthcare professionals annually. It contains concise topic pages providing actionable guidance on clinical matters ranging from health maintenance to rare disease management. Each topic page, created by clinician experts, provides clinical overviews with information on diagnosis, treatment, and referral, as well as contacts for topic subject matter experts, patient education materials and active clinical trials if applicable. Many topics include care process models (CPMs), interactive algorithms containing expandable information boxes with quick overviews on recommended approaches to diagnosis, evaluation, and management. The design is structured to break down complex approaches into manageable steps, utilizing evidence-based recommendations supported by Mayo Clinic experts, such that content represents the synthesis of evidence-based best practices with expert consensus [19,20]. Accuracy of care at Mayo Clinic, including diagnosis and treatment recommendations, has been shown to improve with use of AskMayoExpert compared to other web-based resources [21]. AskMayoExpert care process algorithms are accessible to all Mayo Clinic staff; it is otherwise accessible through individual or other institutional subscriptions.

We produced a CPM with diagnostic and management recommendations, including referral to the Mayo Clinic Chronic Fatigue Syndrome Clinic, published on AskMayoExpert, incorporating the perspective of people with lived experience of ME/CFS. An announcement was made regarding the addition of the ME/CFS topic publication to AskMayoExpert as part of a system-wide weekly email.

### 2.2. Statistical Methodology

The study was approved by the Mayo Clinic Institutional Review Board (#22-008984). The EHR data retrieval program (SlicerDicer) and Excel were used in data processing and retrieval.

The intervention is the introduction of the AskMayoExpert algorithm. The first population sample data was collected before the introduction of the algorithm. The second sample population data was collected after the introduction of the algorithm. In both cases, referrals to the ME/CFS specialty clinic and ultimate specialist diagnosis were measured over a three-month period.

Several codes have been utilized to denote ME/CFS historically. These include “Chronic Fatigue” (ICD R53.82), “Fatigue, Post Viral,” (G93.3), and more recently the “Myalgic encephalomyelitis/chronic fatigue syndrome” (G93.32) subcode, which was released in October 2022 to improve the tracking of patients with ME/CFS. Any of these codes were considered an indication of potential working diagnosis of ME/CFS for this study.

In order to compare results from two subject groups, we utilized basic summary statistics methods. Since referring providers before and after the introduction of the AskMayoExpert are not the same providers, we utilize methods appropriate for two, independent samples of the same population.

Data was rendered binary, with a 0 representing a negative outcome and 1 representing a positive outcome. In this case, a provider utilizing a code that automatically referred a patient to the specialty ME/CFS clinic where the specialty clinic did not diagnose with ME/CFS was considered a 0; if the referral and specialty clinic were in agreement, this was represented using a 1 and termed ‘diagnostic concordance’.

We hypothesized that the introduction of the algorithm would increase the number of referrals to the specialty ME/CFS clinic, and/or the relative number of diagnoses in which the referral’s tentative diagnosis and the specialty clinic’s were concordant.

Summary statistical methods were used to determine the significance of the data, including relative risk, risk ratio, and odds ratio.

Utilizing the sample proportion formula

(1) $$\hat{p}=\frac{n_{1}}{P}$$

we calculated the sample proportions and then the risk difference, orienting our formulae to the positive outcome (concordance).

(2) $$RD=\left({\hat{p}}_{2}-{\hat{p}}_{1}\right)100$$

Finally, we used the relative risk formula, demonstrating by what degree concordance had increased, using the formula

(3) $$RR={\hat{p}}_{2}∕{\hat{p}}_{1}$$

followed by the odds ratio:

(4) $$OR=\;\frac{{\hat{p}}_{2}}{1-{\hat{p}}_{2}}∕\frac{{\hat{p}}_{1}}{1-{\hat{p}}_{1}}$$

A Z Test for comparing two proportions was chosen over McNemar’s test, as these data are two, independent samples of the same population rather than from the same providers at two timepoints.

To calculate the *z*-statistic, we used the pooled proportion ($\hat{p}$), calculated according to the following formula:

(5) $$\hat{p}=\frac{x_{1}+x_{2}}{n_{1}+n_{2}}$$

where $x_{1}$ and $x_{2}$ represent the ‘successes’ in population 1 and 2, respectively—in this case, diagnostic concordance—and n_1_ and n_2_ represent the totals for each sample population. Once we calculated these values, we calculated the z-statistic according to the formula

(6) $$z=\frac{{\hat{p}}_{1}-{\hat{p}}_{2}}{\sqrt{\hat{p}-\left(1-\hat{p}\right)-\left(\frac{1}{n_{1}}+\frac{1}{n_{2}}\right)}}$$

We calculated the *p* value utilizing Fisher’s exact test to provide further evidence of statistical significance; in the formula below, the additive factorial of data in each direction of a standard, two-by-two summary statistics table is divided by the multiplicative factorial of each of the four cells and the total for both samples.

(7) $$p=\frac{\left(a+b\right)!\left(c+d\right)!\left(a+c\right)!\left(b+d\right)!}{\left(a!b!c!d!n!\right)}$$

In order to determine the magnitude of the effect of the intervention, we used Cohen’s *d* statistic, calculated using the absolute value of the difference between the means of the two sample population values, divided by the pooled standard deviation for both samples. To calculate these, we will need the means and standard deviations for each population. We will also need the pooled standard deviation, per the formula

(8) $${SD}_{p}=\sqrt{\frac{\left(n_{1}-1\right)SD_{1}^{2}+\left(n_{2}-1\right)SD_{2}^{2}}{n_{1}+n_{2}-2}}$$

Finally, Cohen’s *d* was calculated using

(9) $$d=\frac{\left|{\bar{x}}_{1}-{\bar{x}}_{2}\right|}{SD_{P}}$$

where $\bar{x}$ represents the mean of each respective independent population, and *SD_p_* is the pooled standard deviation calculated using Formula (8).

In addition, we calculated the confidence interval for these data. Confidence intervals describe how often the true value is expected to fall within that range if the study is repeated within a specific confidence level; we chose 95%. We used the following formula for the confidence interval for the difference between two proportions in independent samples.

(10) $$\left({\hat{p}}_{1}-{\hat{p}}_{2}\right)\pm z_{\frac{a}{2}}\sqrt{\frac{{\hat{p}}_{1}\left(1-{\hat{p}}_{1}\right)}{n_{1}}+\frac{{\hat{p}}_{2}\left(1-{\hat{p}}_{2}\right)}{n_{2}}}$$

Finally, we calculated percent difference between number of concordant diagnoses in each group, and the percent difference in total number of referrals to the specialist ME/CFS clinic over the pre- and post-intervention periods.

For purposes of determining user engagement, we also collected and reported on the number of times the AskMayoExpert algorithm was accessed and by whom, sorting by provider type and by specialty area.

## 3. Results

We hypothesized that introduction of the AskMayoExpert care process algorithm for ME/CFS would be associated with improved concordance between referral to Mayo Clinic’s Chronic Fatigue Specialty Clinic and the specialty clinic’s expert diagnosis of ME/CFS. We also hypothesized that total referrals to the Chronic Fatigue Specialty Clinic would increase.

Data were extracted through the Electronic Health Record SlicerDicer function. Patients seen in the specialty clinic who were identified as having a correlating referral or diagnostic code for ME/CFS were selected within the respective timeframe for analysis (Table 1).

Post-introduction of the AskMayoExpert algorithm, there was a 21.6% greater likelihood that a referral to the ME/CFS specialty clinic and specialty clinic diagnosis was concordant (absolute increase). Referrals were 1.39 times more likely to result in a specialist ME/CFS diagnosis after the introduction of the AME compared to before (relative risk of 1.39) (Table 2).

The odds ratio represents the relative increase in the likelihood of concordance of referral and specialist diagnosis. The odds of response are 1.24 and 3.33, respectively; division of these values produced an odds ratio of 2.69. Relatively speaking, clinicians who referred patients to the specialty ME/CFS clinic after the introduction of the AskMayoExpert algorithm were 2.7 times as likely to achieve referral diagnostic concordance (Table 2).

Given *z* > |1.96|, these groups differ significantly in their incidence of diagnostic concordance with a 95% confidence level; using Fisher’s exact test, *p* value was found to be < 0.01, indicating statistical significance in the difference between both groups (Table 3).

Confidence interval is 0.0408 < p < 0.391, confidence of 95%, indicating that the population difference in proportions is 4.08% to 39.1%. Since these data do not include zero, the difference in concordance in the pre- and post-intervention groups is considered statistically significant (Table 3).

A Cohen’s *d* of ~0.5 represents a moderate impact associated with an intervention. Therefore, this data supports that the introduction of the AskMayoExpert algorithm was associated with a moderate effect on concordance of referral and specialist diagnosis at Mayo Clinic’s ME/CFS specialty clinic (Table 4). For a summary of all calculations, refer to Table 5.

We can only state that increased diagnostic concordance and total referrals to the ME/CFS clinic increased within periods after the AskMayoExpert algorithm’s release. However, to provide additional circumstantial support, we investigated the use of the algorithm by Mayo Clinic providers over the period studied, to demonstrate frequency of access during the study period.

The AskMayoExpert ME/CFS algorithm was accessed 580 times during the study period, with staff consultants, advanced practice providers, and residents/fellows making up the bulk of identified providers (see Table 6). Access was also multidisciplinary, with providers from Internal Medicine, Neurology, Family Medicine, Rheumatology, Geriatrics, Endocrinology, Allergy and Immunology, Psychiatry, Gastroenterology and Hepatology, Physical Medicine and Rehabilitation, Hematology Oncology, Preventative Medicine, Infectious Disease, Anesthesiology/Pain Medicine, Cardiovascular Disease, Orthopedic Surgery, Pediatric Pulmonology/Sleep Medicine, Spine Pediatrics, Pediatric Allergy and Immunology, and Pediatric Clinical Genomics consulting the algorithm outside of the ME/CFS specialty clinic.

## 4. Discussion

**Figure 2:**
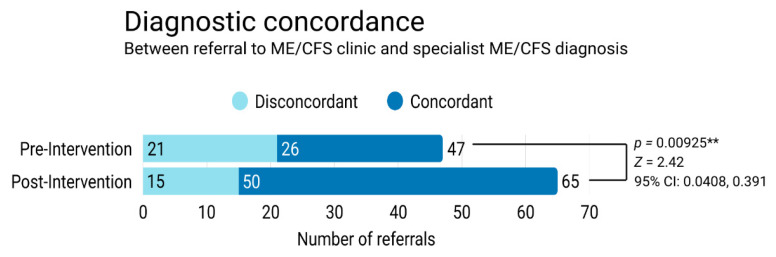
Concordance between referring clinician and ME/CFS specialist diagnosis in a three-month period pre-intervention and a three-month period post-intervention. Both the total number of referrals to the specialist clinic and the percentage of concordant diagnoses increased significantly. ** indicates a *p* value less than 0.01.

The implementation of the AskMayoExpert algorithm was associated with a significant increase in number of referrals to the ME/CFS specialty clinic and increased agreement between referral to the ME/CFS specialty clinic and a specialty clinic’s expert diagnosis of ME/CFS, implying both greater awareness and more accurate knowledge of ME/CFS diagnosis after the implementation of the AskMayoExpert algorithm [Figure 2]. These findings suggest that effective point-of-care resources can improve clinician knowledge and practice with the potential for meaningful impact, as a greater number of patients living with ME/CFS could be diagnosed in a timely manner.

More than one in four individuals with ME/CFS will go at least five years without a formal diagnosis. Reducing diagnostic delay in a portion of patients may result not only in reduced personal harm and costs, but also reduced total system burden from frequent medical appointments and diagnostic testing.

Additionally, it is important to recognize that the AskMayoExpert care process algorithm may be used for reasons other than center referral. Over 12 months, the myalgic encephalomyelitis/chronic fatigue syndrome topic ranked 263 out of 1143 AskMayoExpert topics for number of views, staying above both annual average (298) and median (95) views per topic. Within its own category, it is the second most viewed topic quarterly behind the chronic fatigue (symptom) topic. Our team also received messages relaying appreciation for the information by multiple users. This indicates an interest and need for accessible clinical management information on ME/CFS by clinicians. The diversity of users also reinforces the multidisciplinary involvement of the disease.

We did not gauge the impact for non-referring clinicians in this study. The CPM could have been utilized for care without subsequent referral—suggesting referrals were not required as users found sufficient information to diagnose and manage themselves. This would have been a helpful positive outcome to measure that unfortunately was not feasible based on the non-controlled methods of our study. Although this depth of data was not available at the time of the study, it would have been interesting to see if more individuals referred to the specialty clinic in the later time frames were more likely to have been recommended pacing and/or initial pharmacotherapies as described in the care process algorithm. Answering these questions would require additional user-level data through AskMayoExpert and chart review beyond the permissions granted to the study team, but could be considered for future investigations.

It is important to acknowledge that this was not a controlled study. It is not feasible to know how many of the referring clinicians utilized the algorithm, and it is possible that referring clinicians may have encountered other educational resources on ME/CFS beyond the algorithm. Confounding educational factors were controlled feasibly in that there were no new publications (internally or externally) by the study team on ME/CFS, or educational events on ME/CFS at the study site, during the study period.

This study was conducted at a quaternary specialty center with widespread access to the algorithm. It is possible that our institution’s status as a major academic center increases the likelihood of encountering individuals with ME/CFS, increasing the potential impact of the study intervention where the benefits may not otherwise be as significant in other settings.

One final major limitation was the use of ICD-10 codes to determine diagnostic concordance. Our study places a greater emphasis on working diagnosis of ME/CFS based on use of diagnosis (ME/CFS or chronic fatigue syndrome) over symptom (e.g., fatigue, chronic fatigue) referral codes. While it may be reasonable to suggest greater confidence in referral based on this, we cannot definitively confirm the difference in accuracy of working diagnosis from the referring clinician’s end. This would also require dedicated patient-level chart review and could be a component of future studies. Future studies assessing the impact of an AskMayoExpert CPM or similar development would benefit from matching individual clinicians’ concordance before and after the introduction of the intervention. Extending the study timeline would increase the sample size, supporting its findings more robustly, and demonstrating the general utility of a care process algorithm in diseases where medical education is scant.

A major strength of the study was the composition of the team, which included input from individuals with lived experience, clinical, and research backgrounds in ME/CFS, along with AskMayoExpert build experts. This ensured the inclusion of clinically impactful information in an accessible and effective contact format. The involvement of patients and other people with lived experience is well-supported for effective initiatives particularly in the context of complex chronic conditions [22,23,24]. The diversity of specialty referrals also demonstrates the multilevel impact of ME/CFS.

## 5. Conclusions

Our study evaluated the difference in referral code concordance for ME/CFS following development of a point-of-care diagnostic and management guidance tool through AskMayoExpert. Between publication of the clinical tool and the end of the calendar year, 580 healthcare professionals accessed the tool, referral concordance improved from 55.3% to 76.9%, and referrals to the specialty ME/CFS clinic increased. This suggests that publication of a clinical care algorithm on a widely accessible platform for the institution may assist in improving referring provider recognition of ME/CFS.

## Figures and Tables

**Table 1:** Two-by-two table. Concordance between referral to ME/CFS specialty clinic and specialist diagnosis at Mayo Clinic for three-month periods before and after the introduction of the AskMayoExpert care process algorithm.

	Pre-Intervention (*p*_1_)	Post-Intervention (*p*_2_)	Totals
Concordant diagnosis (1)	26	50	76
Discordant diagnosis (0)	21	15	36
**Totals**	47	65	112

**Table 2:** Summary statistics. Sample proportion, risk difference, risk ratio and odds ratio between pre- and post-intervention data at Mayo Clinic.

	Pre-Intervention (*p*_1_)	Post-Intervention (*p*_2_)	Calculations
Concordant diagnosis (1)	26	50	76
Discordant diagnosis (0)	21	15	36
Totals	47	65	112
$\hat{p}$	0.553191	0.769231	
RD			21.6%
*RR*			1.39
*OR*			2.69

$\hat{p}$ = sample proportions; RD = risk difference; RR = relative risk; OR = odds ratio.

**Table 3:** Z-statistic and *p* values. Sample proportion, difference in proportions (risk difference), risk ratio and odds ratio between pre- and post-intervention data at Mayo Clinic.

	Pre-Intervention (*p*_1_)	Post-Intervention (*p*_2_)	Calculations
Concordant diagnosis (1)	26	50	76
Discordant diagnosis (0)	21	15	36
Totals	47	65	112
$\hat{p}$	$\hat{p}$_1_ = 0.553191	$\hat{p}$_2_ = 0.769231	
$\bar{p}$			0.679
*z*-statistic			2.42
*p* value			9.25 × 10^−3^

$\hat{p}$ = sample proportions; $\bar{p}$ = pooled proportion.

**Table 4:** Cohen’s *d*.

	Pre-Intervention (*p*_1_)	Post-Intervention (*p*_2_)	Calculations
Concordant diagnosis (1)	26	50	76
Discordant diagnosis (0)	21	15	36
Totals	47	65	112
$\hat{p}$	0.553191	0.769231	
$\bar{x}$	0.553191	0.769231	
*SD*	0.502538	0.424604	
*SD_p_*			0.458808
d			0.470871

$\hat{p}$ = sample proportions; $\bar{x}$ = means; *SD* = standard deviation; *SD_p_* = pooled standard deviation; *d* = Cohen’s *d*.

**Table 5:** Summary. Variables comparing pre-intervention to post-intervention concordance.

Variable	Value	Significance Threshold
Risk Difference	0.216	>0
Relative Risk	1.391	>1
Odds Ratio	2.692	>1
z-test	2.416	>1.96
*p* value (Fisher’s exact test)	9.25 × 10^−3^	<0.05
Cohen’s *d*	0.471	0.2
Confidence interval (CI, 95%)	0.0408 < p < 0.391	Must not include zero

**Table 6:** Unique visits to the ME/CFS AskMayoExpert care process algorithm through the end of the study period.

Job Category	Total Unique Views
Staff consultant	220
Advanced practice provider	56
Resident/fellow	27
Other student	1
Registered nurse (RN) or Licensed Practice Nurse (LPN)	14
Pharmacist	9
Healthcare professional/technician	3
Other	46
Unknown	204
**Total**	**580**

## Data Availability

Further data supporting the conclusions of this article may be made available by the authors on request. Requests for data sharing will require review and confirmation through institutional legal processes.

## Abbreviations

The following abbreviations are used in this manuscript:

CPM	Care Process Model
LPN	Licensed Practical Nurse
ME/CFS	Myalgic Encephalomyelitis/Chronic Fatigue Syndrome
PCP	Primary Care Provider
Q	Quarter
RN	Registered Nurse
